# Placental hormone profiles as predictors of preterm birth in twin pregnancy: A prospective cohort study

**DOI:** 10.1371/journal.pone.0173732

**Published:** 2017-03-09

**Authors:** Hui Lim, Sioned Powell, Helen C. Mcnamara, A. Forbes Howie, Ann Doust, Maria E. Bowman, Roger Smith, Jane E. Norman, Sarah J. Stock

**Affiliations:** 1 Tommy’s Centre for Maternal and Fetal Health, MRC Centre for Reproductive Health, University of Edinburgh Queen’s Medical Research Institute, Edinburgh, United Kingdom; 2 Mothers and Babies Research Centre, Hunter Medical Research Institute, John Hunter Hospital, University of Newcastle, Newcastle, New South Wales, Australia; 3 School of Women's and Infants' Health, The University of Western Australia at King Edward Memorial Hospital, Crawley Western Australia, Australia; Academic Medical Centre, University of Amsterdam, NETHERLANDS

## Abstract

**Objective:**

The objective of the study was to analyse placental hormone profiles in twin pregnancies to determine if they could be used to predict preterm birth.

**Study design:**

Progesterone, estradiol, estriol and corticotropin-releasing hormone were measured using competitive immunoassay and radioimmunoassay in serum and saliva samples of 98 women with twin pregnancies,at 3 or more gestational timepoints. Hormone profiles throughout gestation were compared between very preterm (<34 weeks; n = 8), preterm (<37 weeks; n = 40) and term (37+ weeks; n = 50) deliveries.

**Results:**

No significant differences were found between preterm and term deliveries in either absolute hormone concentrations or ratios. Estimated hormone concentrations and ratios at 26 weeks did not appear to predict preterm delivery. Salivary and serum hormone concentrations were generally poorly correlated.

**Conclusion:**

Our results suggest that serial progesterone, estradiol, estriol and corticotropin-releasing hormone measurements in saliva and serum are not robust biomarkers for preterm birth in twin pregnancies.

## Introduction

Multiple pregnancies result in significant neonatal mortality five to six times greater than that of singletons [1]. Preterm birth (PTB) is the biggest single contributing factor to this. More than half (56%) of multiple pregnancies deliver before 37 weeks’ gestation, compared to just 6% of singletons [2]. Compared to singletons, surviving twins experience a higher risk of respiratory distress, hypothermia, seizures and rehospitalization, as well as motor and cognitive problems in childhood [3].

The lack of adequate screening methods or preventative measures for those multiple pregnancies at high risk of spontaneous PTB presents a major challenge. While mechanisms such as increased uterine stretch, elevated placental corticotropin-releasing hormone (CRH), fetal lung factors, infection, stress, cervical insufficiency and placental dysfunction have been proposed to explain the higher PTB rate in multiple pregnancies, the relative contribution of each of these is unknown [4].

Differences in the efficacy of PTB prevention strategies between singleton and twin pregnancies also suggest underlying mechanistic differences. Progesterone (P4) administration reduces PTB in singletons [5] but not twins [6]. In PTB predictive tests, different cut-off values of cervical length (CL) and fetal fibronectin (FFN) are used for singletons and twins, possibly reflecting differing pathophysiological mechanisms causing the onset of spontaneous PTB.

A previous study [7] proposed that a single measurement (>2.1 ng/ml) of salivary estriol (E3) could predict an increased risk of spontaneous PTB in singleton pregnancies. Three later studies, two in saliva and one on serum, suggested that repeating measurements of hormones to generate a “hormone profile” may be more useful than a single measure [8–10]. Priya *et al*. and Lachelin *et al*. found that low saliva P4 between 24–34 weeks was associated with spontaneous PTB before 34 weeks [8,9]. Smith *et al*. found a significantly higher daily percentage change in CRH at 26 weeks in preterm than term deliveries, and increased E3:E2 and decreased P4:E3 ratios in the month before delivery [10]. Given this evidence, it would appear that P4, E2, E3 and CRH profiling may be useful in predicting PTB. However, as these studies were performed predominantly in singleton pregnancies (Smith *et al*. included 13 women with multiple pregnancies in their cohort of 500 women), the potential for hormone profiles to predict PTB of twins is unknown. Our aim was to ascertain whether the time trends identified in singletons (increased E3:E2 and decreased P4:E3 ratios in the month before delivery) applied in twins, and if gestational hormone profiles could help predict PTB of twins.

We hypothesized that hormone profiles of P4, E2, E3 and CRH are different in twin pregnancies delivering preterm and at term and may serve as reliable markers for preterm birth and/or spontaneous preterm birth in twin pregnancies. Both serum and saliva have been previously used to determine concentrations of steroids in pregnancy. Saliva is gaining increasing recognition as a good alternative with diagnostic value [11], as it contains serum constituents that reach the oral cavity via blood vessels and gingival fluid [12]. Steroids are highly stable in saliva [13] and salivary sex steroids have been used in clinical settings to assess fertility and ovarian function. Besides representing free and biologically-active hormone concentrations, salivary specimen collection is non-invasive and convenient. Subjects can collect samples themselves as often as required and storage and transportation are convenient as thawing and re-freezing cycles do not affect assays. A secondary aim was thus to compare serum and salivary hormone profiles to determine if either was superior in prediction of preterm birth in twins.

## Materials and methods

Ethical approval for the study and consent procedure was from the Lothian Regional Ethics Committee (reference S1103) and the West of Scotland REC for the Edinburgh Reproductive Tissue BioBank (reference 09/50704/3). Written and verbal information was provided to participants and written informed consent was obtained by the clinician involved in their care on an approved consent form.

Women were enrolled from the specialist multiple pregnancy antenatal clinic at The Simpson’s Centre for Reproductive Health (SCRH), Royal Infirmary of Edinburgh between April 2010 and September 2011. The SCRH is a teaching hospital and tertiary referral center with approximately 7000 deliveries per annum. All women with multiple pregnancies booked to deliver in the hospital are cared for in a specialist multiple pregnancy clinic. Eligible women were identified at their first clinic visit (usually at 11–16 weeks’ gestation) and invited to participate. Informed consent was obtained by the clinician involved in their care. Five ml of blood was collected via venepuncture and saliva was collected using Salivette kits (Sarstedt AG & Co, Nümbrecht, Germany) at each clinic attendance. The Salivette kits used (51.1534) contain an untreated cotton swab in a collection tube. Saliva collections was performed as per manufacturers instructions—saliva is stimulated by chewing on a cotton swab for 60 seconds, returned to the Salivette collection tube, and centrifuged for 2 minutes at 1,000 x g, with clear saliva collected in sample tube, and particles and mucus strands collected in the tip of the Salivette tube. Women were asked to refrain from eating for 30 minutes before sample collection. Sample collection was pragmatic, with samples requested whenever women attended clinic for consultation. For most participants visits were scheduled at 16, 20, 28, 32 and 36 weeks’ gestation.

### Inclusion and exclusion criteria

Inclusion criteria were age of ≥16, twin pregnancy with chorionicity and gestation established on ultrasound scan before 16 weeks’ gestation, gestation of less than 20 weeks at recruitment, no known serious congenital abnormality and intending to deliver at the SCRH. Subjects who tested positive for HIV, Hepatitis B or C or who were on progesterone therapy were excluded. Further pre-specified exclusion criteria were applied after sample collection. As an aim was to assess hormone profiles and interpolated values from 26 weeks gestation, subjects with gestational length less than 26 weeks or fewer than 3 blood or 3 saliva measurements were excluded from analysis, in accordance with previously published criteria [10].

### Clinical data

Clinical data was collected prospectively from electronic patient records (TrakCare) and recorded on case report forms. Pregnancy outcome data included gestation at delivery, preterm delivery (<37 weeks’ gestation), very preterm delivery (<34 weeks’ gestation), labor onset (spontaneous or iatrogenic), pregnancy complications and maternal medical conditions (pre-eclampsia, suspected fetal growth restriction, diabetes, other), delivery method and birth weight. Potential confounding factors collected included age, body mass index (BMI), parity and smoking status.

### Hormone measurement

Samples were centrifuged at 4°C, 3000g for 15 minutes, with supernatants aliquoted and stored at -80°C until processing. Saliva and serum P4, E2 and E3 were measured by competitive immunoassay using commercial Enzyme-Linked Immunosorbent Assay kits (Demeditec Diagnostics GmbH, Kiel, Germany; Total E2 DE3355, Saliva E2 DESLV4188, Total P4DE1561, Saliva P4 DES6633, Total E3 DE3717, Saliva E3 DES6644) according to manufacturer’s protocol. Analytical sensitivity of assays were 0.045ng/ml, 9.714pg/ml and 0.22ng/ml for serum P4, E2 and E3 respectively, and 5.0pg/ml, 0.4pg/ml and 1.0pg/ml for saliva P4, E2 and E3 respectively. Where sample hormone concentrations exceeded the assay dynamic range, they were diluted with charcoal-stripped human serum and incubation buffer for serum and saliva samples respectively. All kits performed as reported by the manufacturer, with intra- and inter-assay coefficients of variation (CV) of under 6% and approximately 8% respectively across the working range. CRH assays were performed on two plasma samples per subject using radioimmunoassay according to the previously published protocol [10]. Assays were conducted blind to pregnancy outcome and samples were analyzed in duplicates, with intra- and inter-assay CV of 7.6% and 9.8% respectively.

### Statistical analysis

Sample size was calculated based on differences in median concentrations of CRH in singleton pregnancies that deliver at term and preterm (5pmol/L with a standard deviation of 4.296) [10]. We calculated that a sample size of 70 (to achieve 10 spontaneous PTBs before 34 weeks and 51 deliveries after 34 weeks) would have 95% power to detect this difference. Interim assessment of recruitment revealed fewer women delivered spontaneously preterm than anticipated, and fewer women with preterm deliveries donated three samples, thus recruitment was continued to a total of 121 to ensure adequate power. All statistical analyses were performed using SPSS Statistics 22.0. Curve-fitting was used to obtain hormone trajectories, a single smoothed curve representative of hormone concentrations across gestation of a number of subjects. Our previously described hormone-specific equations (S1 Table Supplementary) were used to transform trajectories into linear functions, under the assumption that a single equation type could be used to curve-fit samples for each analyte [10]. Graphs were visually checked for linearity to confirm the general validity of these equations. 26-week concentrations derived from hormone trajectories were used for subsequent analyses.

Hormone concentrations, hormone ratios (P4:E2, P4:E3 and E3:E2) and derived variables of CRH (rate, daily percentage change) at 26 weeks were compared in women who delivered very preterm (<34 weeks’ gestation), preterm (<37 weeks’ gestation) and at term (37+ weeks’ gestation). The Mann-Whitney U test was used to identify significant differences. Differences in variables were compared across the three groups with one-way ANOVA for continuous variables and chi-square for categorical variables. To test PTB prediction potential, the relationship between 26-week hormone concentrations and gestation at delivery was determined by Spearman’s rank correlation coefficient analysis. Salivary and serum hormone concentrations were also compared with Spearman’s coefficients.

### Subgroup analysis

Similar but separate analyses were conducted for spontaneous PTBs, with medically indicated preterm deliveries excluded.

### Missing data

**Fig 1:**
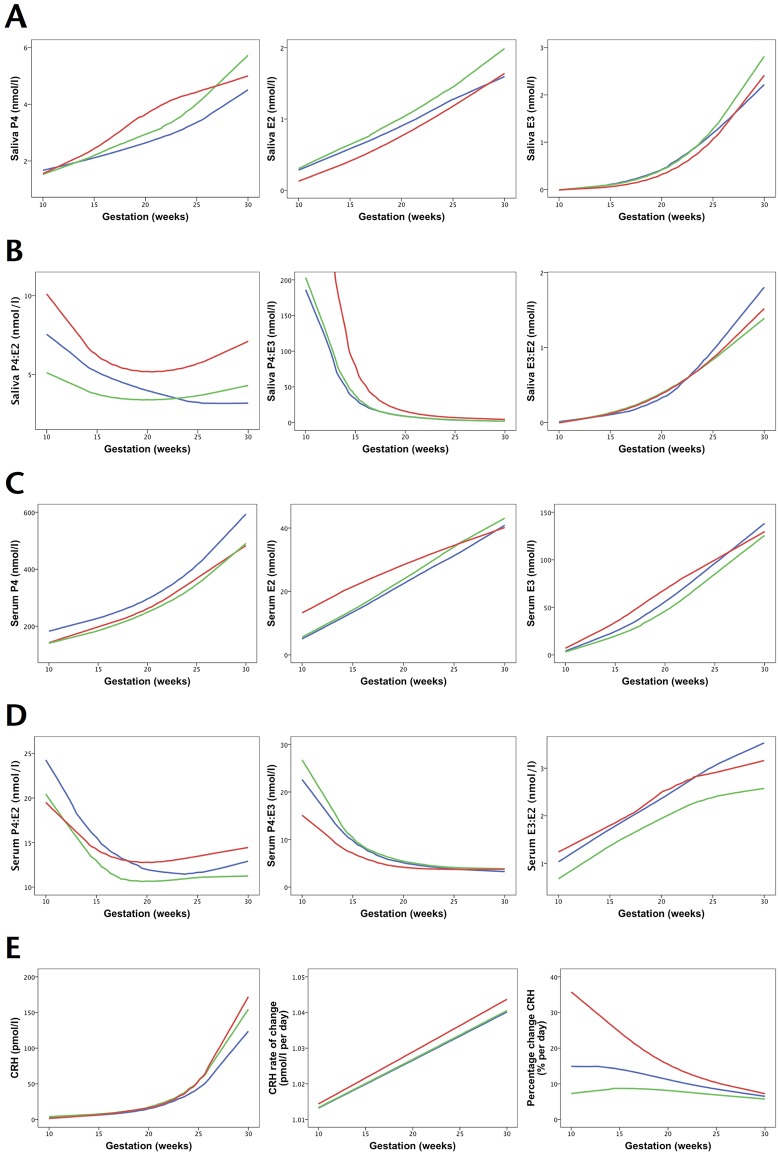
Hormone trajectories (smoothed median curves) in early preterm (n = 8), preterm (n = 40) and term (n = 50) groups. *Red lines*, <34 weeks at delivery; *green lines*, 34-<37 weeks; *blue lines*, 37+ weeks. (**A**) Salivary concentrations (n = 7, 35, 43 for <34, 34-<37 and 37+ weeks respectively). (**B**) Salivary ratios. (**C**) Serum concentrations (n = 8, 41, 43 for <34, 34-<37, 37+ weeks respectively). (**D**) Serum ratios. (**E**) CRH concentrations and derived variables.

We pre-specified that women with fewer than 3 samples (n = 16), or missing delivery data (n = 2) would be excluded. Hormone trajectories were modeled for each woman with ≥3 samples, based on available hormone measurements (Fig 1).

## Results

Two hundred and six women attended the multiple pregnancy clinic during the study period, of whom 43 were ineligible for participation at the time of screening (20 being more than 20 weeks gestation; 23 with other exclusion criteria). Forty-two women declined participation and 121 women were recruited to the study. Twenty- three women were subsequently excluded due to withdrawing midway (n = 4), having incomplete delivery data due to transfer to another hospital (n = 2), delivery or fetal demise at less than 26 weeks’ gestation and/or having fewer than three samples available for analysis (n = 17). This left a cohort of 98 women with clinical and hormonal data for analysis.

Eight of 98 women delivered very preterm, 40 delivered preterm and 50 delivered at term. Clinical details of the three groups are provided in Table 1. The groups were similar with respect to maternal age, BMI, parity, smoking status and chorionicity (Table 1).

**Table 1:** Maternal and fetal characteristics and pregnancy outcomes classified as early preterm, preterm and term deliveries.

Study cohort (n = 98)	Early Preterm (<34 weeks)	Preterm (34–36+6 weeks)	Term (>37 weeks)	*p* values
n	8 (8%)	40 (41%)	50 (51%)	
**Chorionicity**				
Dichorionic	7 (88%)	27 (68%)	42 (84%)	0.14
Maternal age (years)	33 ± 7.0	32 ± 5.4	33 ± 5.0	0.59
Maternal BMI (kg/m^2^)	24 ± 3.4	27 ± 5.9	25 ± 4.4	0.13
**Parity**				
Primiparous	8 (50%)	25 (63%)	24 (49%)	0.41
**Smoking, self-reported**				
Prior to pregnancy	3 (30%)	13 (33%)	15 (30%)	0.35
Current	0 (0%)	6 (15%)	3 (6%)	0.22
**Outcomes**				
**Complications**	2 (25%)	21 (53%)	5 (10%)	0.00*
Pre-eclampsia or PIH	1	9	1	-
IUGR	1	5	0	-
Others	0	7	4	-
None	6 (75%)	19 (48%)	45 (90%)	0.09
Gestational age at delivery (weeks)	33 ± 1.30	36 ± 0.92	38 ± 0.62	0.00*
**Labor onset**				
Spontaneous	6 (75%)	11 (28%)	6 (12%)	0.00*
Induction	0	9 (23%)	13 (26%)	0.26
Caesarean section	2 (25%)	20 (50%)	31 (62%)	0.12
**Delivery method**				
Caesarean section	6 (75%)	32 (80%)	37 (74%)	0.68
Vaginal birth	2 (25%)	7 (18%)	13 (26%)	0.62
Vaginal, then caesarean	0	1 (3%)	0	-
Estimated blood loss (ml)	721 ± 378	788 ± 477	729 ± 490	0.83
**Birth weight (g)**				
Twin 1	1964 ± 203	2363 ± 365	2775 ± 355	0.00*
Twin 2	2000 ± 303	2352 ± 350	2669 ± 426	0.00*

*PIH*, pregnancy-induced hypertension. *p* values obtained using one-way ANOVA for continuous variables, chi-squared tests for categorical variables.

*p < 0.001.

A total of 475 serum and 453 saliva samples from 98 subjects were analyzed, with the median number of samples provided by each woman being four (range: 3–9) saliva and four (range: 3–9) serum.

### Normal hormone concentrations and trajectories in twin pregnancies

Previously described equations [10] (S1 Table) were suitable for curve-fitting, as evidenced by the post-transformation linear graphs of hormone concentrations in 45 uncomplicated, full-term twin pregnancies (S1 Fig).

### Preterm vs term comparisons

To compare preterm and term profiles, smoothed median curves of hormone concentrations and ratios were used to illustrate hormone trajectories across gestation. On visual inspection, trajectories in early preterm, preterm and term pregnancies were found to be largely similar (Fig 1).

Estimated hormone concentrations and derived variables for each group were interpolated at 26 weeks and compared. No significant differences were found between estimated P4, E2 and E3 concentrations and P4:E2, P4:E3 and E3:E2 ratios at 26 weeks in saliva and serum when comparing preterm or early preterm pregnancies with term. The same was true for CRH level, rate and percentage change. All asymptotic significance values exceeded 0.05 (Table 2).

**Table 2:** Median derived values of hormones at 26 weeks gestation in early preterm, preterm and term births, and comparisons between early preterm and term births, and preterm and term births.

	Derived 26 week median hormone concentrations (nmol/l) and ratios (all deliveries)			Preterm vs Term comparisons			
	Early Preterm (<34 weeks)	Preterm (34–36+6 weeks)	Term (>37 weeks)	Preterm vs Term		Very preterm vs Term	
	n = 8	n = 40	n = 50	MWU Value	p value	MWU Value	p value
**Saliva**							
P4	4.58	4.16	3.51	790	0.141	192	0.158
E2	1.27	1.57	1.36	90	0.446	12	0.773
E3	1.22	1.47	1.37	211	0.899	17	0.773
P4:E2	5.83	3.72	2.98	106	0.933	2	0.149
P4:E3	6.04	3.11	2.82	157	0.214	4	0.176
E3:E2	0.96	0.939	1.15	49	0.478	10	0.88
**Serum**							
P4	437.86	365.34	387.93	1123	0.582	144	0.242
E2	35.72	36.54	33.33	1166	0.806	182	0.761
E3	105.62	94.78	105.93	988	0.175	184	0.851
P4:E2	13.74	11.09	11.37	1125	0.592	167	0.52
P4:E3	3.7	3.88	3.65	1032	0.299	174	0.688
E3:E2	2.91	2.49	3.19	1026	0.279	192	1
**CRH**							
CRH level	1.784	1.785	1.698	807	0.077	162	0.13
CRH rate	0.016	0.015	0.015	1004	0.842	167	0.16
CRH % rate of change	0.977	0.822	0.903	867	0.197	228	0.948

MWU, Mann-Whitney U.

A similar analysis restricted to spontaneous PTB (excluding iatrogenic PTBs) was conducted and found no significant differences in hormone concentrations and ratios between spontaneous PTBs and normal term deliveries. Asymptotic significance values are reflected in Table 3.

**Table 3:** Median derived values of hormones at 26 weeks gestation in spontaneous early preterm, spontaneous preterm and term births, and comparisons between spontaneous early preterm and term births, and spontaneous preterm and term births.

	Derived 26 week median hormone concentrations (nmol/l) and ratios (spontaneous preterm births)			Spontaneous Preterm vs Term comparisons			
	Sp Early PTB (<34 weeks)	Sp PTB (34–36+6 weeks)	All Term (>37 weeks)	Preterm (n = 17) vs Term (n = 50)		Early preterm (n = 6) vs Term (n = 50)	
	n = 6	n = 11	n = 50	MWU Value	p value	MWU Value	p value
**Saliva**							
P4	4.4	3.91	3.51	310	0.351	94	0.208
E2	-	1.16	1.36	10	0.313	-	-
E3	-	1.54	1.37	9	0.677	-	-
P4:E2	-	4.7	2.98	8	0.208	-	-
P4:E3	-	2.89	2.82	12	1	-	-
E3:E2	-	1.43	1.15	3	0.329	-	-
**Serum**							
P4	390.28	385.77	387.93	409	0.912	137	0.787
E2	35.72	31.57	33.33	378	0.572	145	0.957
E3	105.62	94.23	105.93	311	0.148	127	0.64
P4:E2	13.74	12.22	11.37	368	0.477	128	0.608
P4:E3	3.7	5.07	3.65	321	0.194	128	0.66
E3:E2	2.91	2.82	3.19	374	0.612	137	0.847
**CRH**							
CRH level	1.784	1.744	1.698	384	0.406	139	0.226
CRH rate	0.016	0.016	0.015	388	0.44	156	0.428
CRH % rate of change	0.977	0.834	0.903	432	0.896	185	0.932

MWU, Mann-Whitney U. -, insufficient data.

### Predictive value of hormone profiles

The ability of hormone profiles to predict preterm delivery was assessed using Spearman’s rank correlation coefficients. All values were non-significant (Table 4), indicating that no salivary or serum hormone level or derived variable correlated with the eventual time of delivery. Ultimately, none of the absolute values or ratios of hormones investigated were able to predict PTB.

**Table 4:** Spearman’s correlation coefficient analysis to determine the relationship between predicted hormone concentrations at 26 weeks’ gestation and ultimate gestation at delivery.

All deliveries	Median concentrations (nmol/l) and ratios			Spearman’s correlation coefficient analysis	
	Very Preterm (<34) n = 8	Preterm (34-<37) n = 40	Term (37+) n = 50	Spearman’s rho	p value (2-tailed)
**Saliva**					
P4	4.58	4.16	3.51	-0.128	0.233
E2	1.27	1.57	1.36	-0.214	0.257
E3	1.22	1.47	1.37	-0.076	0.634
P4:E2	5.83	3.72	2.98	0.131	0.489
P4:E3	6.04	3.11	2.82	-0.114	0.479
E3:E2	0.96	0.939	1.15	0.174	0.426
**Serum**					
P4	437.86	365.34	387.93	0.021	0.836
E2	35.72	36.54	33.33	-0.051	0.618
E3	105.62	94.78	105.93	0.113	0.272
P4:E2	13.74	11.09	11.37	0.069	0.498
P4:E3	3.7	3.88	3.65	-0.069	0.504
E3:E2	2.91	2.49	3.19	0.095	0.354
**CRH**					
CRH level	1.784	1.785	1.698	-0.159	0.132
CRH rate	0.016	0.015	0.015	-0.052	0.626
CRH % rate of change	0.977	0.822	0.903	0.092	0.384

### Saliva vs serum comparisons

Salivary hormones represent the metabolically-active, free and unbound fraction of total serum concentrations. To find out if salivary and serum data are congruous, we compared concentrations in several individuals as well as overall trends in the entire cohort.

**Fig 2:**
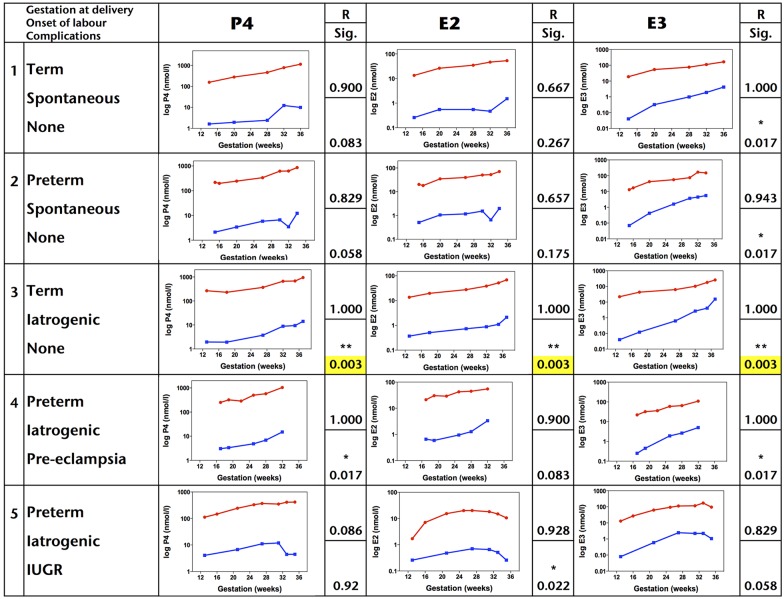
Correlation of salivary and serum hormone concentrations in five individuals. *Red lines*, serum concentrations; *blue lines*, saliva concentrations.*R*, Spearman’s correlation coefficient (rho) value. *Sig*., significance value. *, *p* < 0.05; **, *p* < 0.01.

To obtain a comparison of salivary and serum hormone concentrations in an individual, we selected five subjects with different gestations at delivery, mode of labor onset and complications and plotted their P4, E2 and E3 concentrations throughout gestation (Fig 2). Serum concentrations were consistently higher than salivary concentrations. Of the three hormones, E3 was found to be the most correlated in saliva and serum, exhibiting a significant correlation in 4 of the 5 subjects.

To compare salivary and serum concentrations across the cohort, 26 week concentrations of each hormone and ratio were interpolated and compared using the Spearman’s correlation coefficient analysis. Only P4 and the P4:E2 ratio were found to have a positive correlation in terms of their salivary and serum concentrations (*p* = 0.042 and *p* = 0.002 respectively) (Table 5).

**Table 5:** Spearman’s correlation coefficient analysis comparing salivary and serum concentrations.

Predicted hormone concentrations at 26 weeks	P4	E2	E3	P4:E2	P4:E3	E3:E2
n	85	30	40	30	39	23
Spearman’s rho	0.221	0.332	0.053	0.536	0.016	0.190
p value (2-tailed)	0.042*	0.073	0.744	0.002**	0.921	0.386

## Discussion

We sought to determine whether placental hormone profiles could be used as predictive biomarkers of PTB in twin pregnancies. Our data suggests that serial measurements of hormones in saliva and serum do not function as markers of preterm or very preterm birth of twins.

The curve-fitting equations described in a previous paper [10] based on full-term, spontaneous singleton deliveries were applicable to our data and adequately described term and preterm twin pregnancies. Both the previous and current studies found no significant differences in estimated 26-week concentrations and ratios between preterm and term deliveries, suggesting that any preterm-term differences in hormone concentrations are not large enough to distinguish them. The lack of predictive value in CRH and its derivatives indicated by our data contrasts with the predictive role of percentage change in CRH suggested by the previous paper in singletons, potentially representing the mechanistic differences between twins and singletons. Both studies had similar exclusion criteria and similar statistical methods [7] were employed as far as possible to make our findings directly comparable. We attempted to increase assay uniformity by conducting CRH measurements in the same laboratory using the same method.

In contrast to our findings, Lachelin *et al*. 2009 [9] found low saliva P4 to be a predictor of spontaneous early PTB in women with higher PTB risk. Comparing our data with theirs suggests that firstly, saliva P4 is significantly higher in twin pregnancies than in singletons and secondly, saliva P4 was lower in very preterm than term deliveries in singletons but not in twins. This again suggests that there are intrinsic differences between twin and singleton pregnancies with regards to hormone trajectories. This observed singleton-twin difference may be due to higher median hormone concentrations in twins making preterm-term differences less evident. Median CRH concentrations have been found to be 3.4 times higher in twins [10], potentially explaining the difference in ability of CRH to predict PTB of singletons and twins.

Our attempt to identify a predictive marker of PTB was based on blood and saliva samples obtained on clinic attendance. This study design has several strengths. This practical and cost-effective approach tests a sampling method that is feasible and easily adaptable for clinical use. The use of standard commercial kits according to the manufacturer’s protocol for hormone level measurement ensures minimal inter-center variation. Prospective enrolment of subjects allows a more reliable assessment of serial hormone measurements as a population-screening tool. The demographics of this cohort are those of the Scottish Caucasian population, with an average BMI of 25.4 kg/m^2^, whilst the PTB rate (49.0%) was slightly lower than that reported in the National Centers for Health Statistics’ Vital Statistics Data files in the USA in 2013 (56.6%) [14].

Nevertheless, the study has some limitations. Pragmatic sample collection during clinic attendance may have introduced a selection bias as some subjects may have required more appointments or been more compliant in attendance, hence contributing more samples. It also meant that the exact gestational ages at which samples were obtained could not be standardized for all subjects. Our study was potentially limited by the high incidence of iatrogenic preterm birth and the low number of spontaneous preterm deliveries (n = 23). Nevertheless, this is broadly comparable to previous, larger-scale studies [14], and a post-hoc power calculation found our study had greater than 90% power to determine the difference in hormone concentrations between both very preterm(<34 weeks’ gestation) and term, and preterm(<37 weeks’ gestation) and term deliveries.

We observed a generally low correlation between salivary and serum hormone concentrations, suggesting that they cannot act as substitutes of one another. This could be due to varying modes and extents of hormone transport from blood to saliva. Transport could occur via passive diffusion or active transport and salivary concentrations may be further influenced by reabsorption or dynamic changes in transport proteins nearing the time of delivery [15]. Proposed mechanisms have thus far been speculative with no specific molecules or carrier proteins identified.

A study of the saliva-serum relationship of sex steroids in singleton pregnancies showed moderate but statistically significant correlations for P4, E2 and E3 (*Pearson’s r = 0*.*65*, *0*.*71*, *0*.*71* respectively, n = 28) in women with singleton pregnancies at 32–38 weeks’ gestation [16]. This contrasts with our data in twins. The low correlation observed in our data may once again represent differences in hormonal mechanisms between singleton and twin pregnancies. Changes in concentrations of binding proteins e.g. sex hormone-binding globulin, corticosteroid-binding globulin may differ, resulting in lower correlation in twins. Different statistical approaches to analyses in the two studies may also contribute to differences.

Our study provides the first detailed investigation of placental hormone profiles in twin pregnancies. It has gathered a sizeable amount of valuable clinical and hormonal data on twin pregnancies upon which further studies and analyses can be based upon. The study also attempts to explore the complexities of investigating twin pregnancies as opposed to singletons and the nature of the saliva-serum hormone relationship.

Further research should focus on elucidating singleton-twin differences in placental hormones and the mechanisms involved in twin PTB. Potential predictive tests may benefit from including known clinical risk factors e.g. previous PTB in addition to biochemical measurements. Taking the clinical history into consideration in the development of a predictive test may better account for the multifactorial nature of the mechanisms underlying PTB.

## Conclusions

While we are unable to conclude that hormone profiles in preterm and term twin births are similar, it is evident that any differences cannot be easily identified and harnessed as a diagnostic tool. Significantly higher median hormone concentrations in twin pregnancies may mask subtle changes and make hormone profiling less robust. Our findings may also suggest some singleton-twin differences in hormonal mechanisms triggering delivery. In isolation, serial hormone measurements in saliva or serum are unlikely to work as an effective predictive tool.

## Supporting information

S1 Table Equations for curve-fitting based on serum hormone concentrations [10]. (DOCX)

S1 Fig Smoothed median curves of hormone concentrations in uncomplicated term pregnancies (n = 45). (**A**) Salivary concentrations (n = 43). (**B**) Salivary ratios. (**C**) Serum concentrations (n = 43). (**D**) Serum ratios. (**E**) CRH concentrations and derived ratios (n = 42).
(TIF)

S1 File Raw data file. (XLSX)
