# Supplementary material for: Placental hormone profiles as predictors of preterm birth in twin pregnancy: A prospective cohort study
Source: PLoS One. 2017 Mar 9;12(3):e0173732. doi: 10.1371/journal.pone.0173732 (PMC5344513; doi:10.1371/journal.pone.0173732)
Supplement: S1 Table — (DOCX) [file pone.0173732.s001.docx]

**S1 Table** Equations for curve-fitting based on serum hormone levels^9^

| **P** | log_e_P = a + bt^1.5^ |
| --- | --- |
| **E2** | log_e_E2 = a + b/(log t) |
| **E3** | log_e_E3 = a + b/ √t |
| **CRH** | log_e_CRH = a + bt^2^ |

t = gestational age in days
