# Supplementary figures and images for: Placental hormone profiles as predictors of preterm birth in twin pregnancy: A prospective cohort study
Source: PLoS One. 2017 Mar 9;12(3):e0173732. doi: 10.1371/journal.pone.0173732 (PMC5344513; doi:10.1371/journal.pone.0173732)

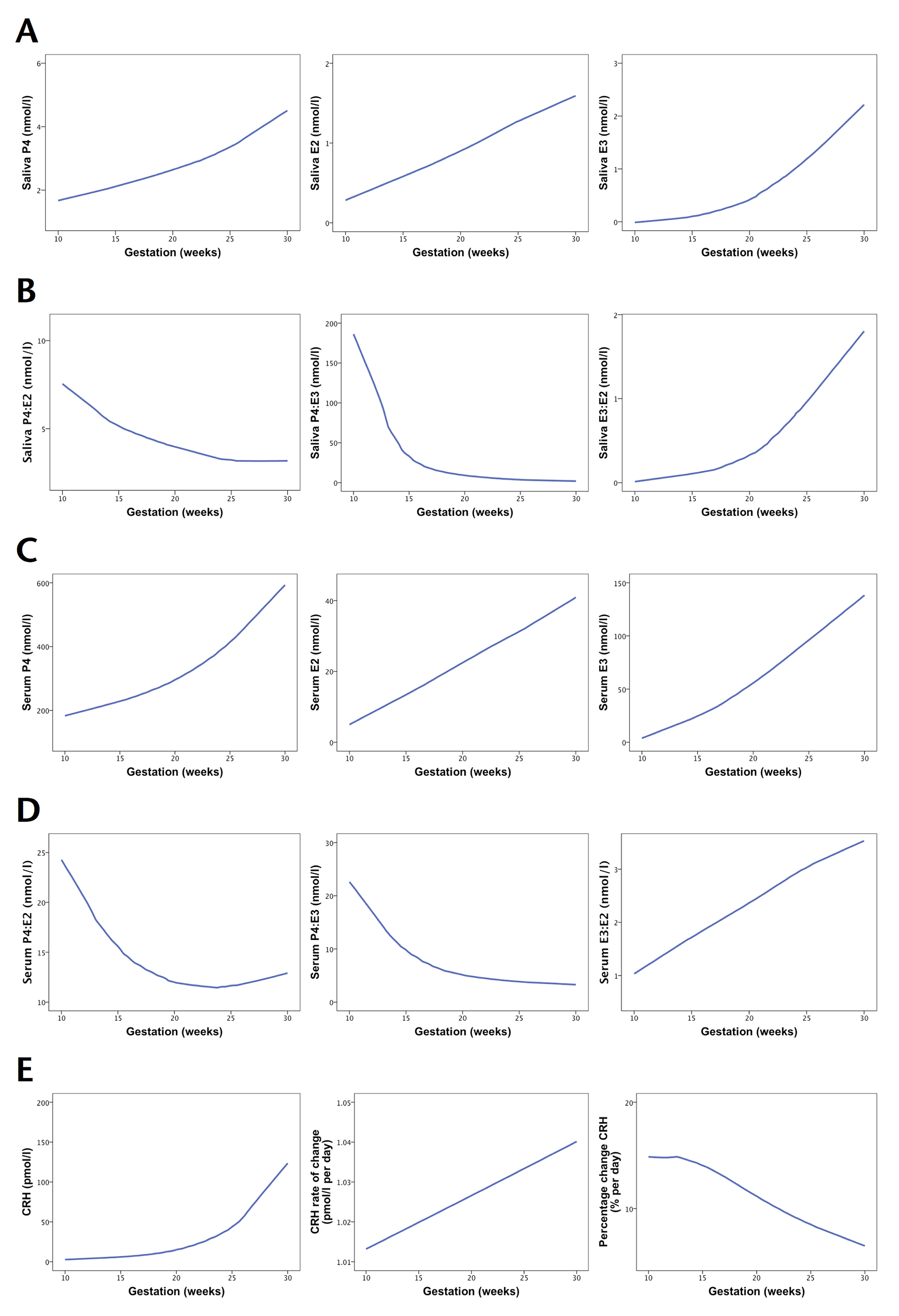

Supplement: S1 Fig — (A) Salivary concentrations (n = 43). (B) Salivary ratios. (C) Serum concentrations (n = 43). (D) Serum ratios. (E) CRH concentrations and derived ratios (n = 42). (TIF) [file pone.0173732.s002.tif]
